# Toll Like Receptor 4: A Potential Link Between Obesity and Metabolic Diseases

**DOI:** 10.1111/obr.70107

**Published:** 2026-02-16

**Authors:** Ghadeer Alhamar, Joanna Razafiarison, Fawaz Alzaid, Fahd Al‐Mulla, Rasheed Ahmad

**Affiliations:** ^1^ Department of Immunology and Microbiology Dasman Diabetes Institute Kuwait City Kuwait; ^2^ INSERM UMR‐S1151, CNRS UMR‐S8253 Université Paris Cité, Institut Necker Enfants Malades Paris France; ^3^ Department of Bioenergetics and Neurometabolism Dasman Diabetes Institute Kuwait City Kuwait; ^4^ Department of Translational Research Dasman Diabetes Institute Kuwait City Kuwait

**Keywords:** inflammation, obesity, TLR, type 2 diabetes

## Abstract

Epidemiological evidence shows that obesity increases the risk of developing metabolic diseases. Nevertheless, the mechanisms behind this connection remain underappreciated. The substantial impact of these disorders on global health has led to extensive research efforts aimed at identifying the pathophysiological links between them. Chronic low‐grade inflammation, induced by altered secretion of adipokines and other bioactive molecules, from adipose tissue, is believed to causally link obesity to various metabolic disorders. Multiple studies have indicated that TLR4 regulates inflammation, adipogenesis, thermogenesis, and glucose metabolism through its interaction with endotoxins, particularly in the context of obesity. The increased expression of TLR4 observed in obesity is believed to contribute to the development of type 2 diabetes (T2D), as it disrupts key physiological processes that regulate metabolic inflammation. This review aims to summarize recent research on the pathobiological roles of TLR4‐mediated inflammation in obesity and its contribution to the development of metabolic disorders. Overall, current evidence supports a central role for TLR4 as a mediator of obesity‐associated metabolic inflammation, highlighting TLR4 and its downstream pathways as promising targets for preventing or treating obesity related metabolic diseases.

## Graphical abstract

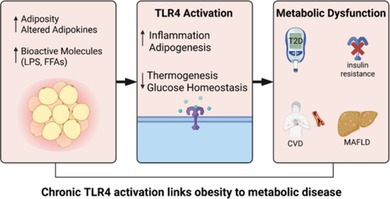

## 1 Introduction

Obesity and metabolic disorders are a growing global epidemiological concern. Obesity is a majorly complex multifactorial disorder, which has been shown to be a risk factor for numerous metabolic disorders, including type 2 diabetes (T2D) [1], metabolic dysfunction associated steatotic liver disease (MASLD) [2], and metabolic syndrome. The increasing prevalence of these conditions is associated with poorer lifestyle choices, which includes but is not limited to higher increases in calorie‐dense diets and more sedentary life choices. Other factors attributing to obesity include certain genetic factors [3], which can affect appetite and cravings, metabolism, and body fat distribution [4], as well as environmental factors, and socioeconomic status [5]. The pathophysiology of obesity has been associated with chronic low‐grade inflammation in adipose tissue because of consistent overnutrition, leading to a defective immune system and hormonal imbalances [6].

The innate immune system plays its most well‐known role as the first line of defense against invading pathogens. Years of research have gone into resolving mechanisms by which the innate immune system recognizes and targets microbes. The discovery of toll like receptors (TLRs) in the mid‐1990s, which demonstrated that pathogen recognition was specific, with a reliance on germline encoded pattern recognition receptors (PRRs) that are able to identify and detect exogenous molecules on the surface of pathogens, deemed pathogen‐associated molecular patterns (PAMPs), or endogenous markers released during tissue damage, called damage‐associated molecular patterns (DAMPS) [7, 8]. TLRs are a group of type 1 transmembrane proteins, with leucine rich repeats at their ectodomains that allow for the recognition of PAMPs. Currently, 10 human and 12 mouse TLRs have been characterized, with TLRs 1–9 being conserved among both species. Table 1 shows a summary of the 10 human TLRs.

**TABLE 1:** Overview of Toll‐Like Receptors 1–10: Function, Cellular Expression, and Dimerization.

	Function	Expression	Dimerization	References
TLR1	Recognizes bacterial lipoproteins from various microbes.	Monocytes, macrophages, dendritic cells, lymphoid tissue.	Forms heterodimers with TLR2	[9, 10]
TLR2	Identifies lipoproteins, peptidoglycan, and lipoteichoic acid from gram‐positive bacteria and fungi.	Monocytes, macrophages, dendritic cells, and to a lesser extent T cells, B cells, and NK cells.	Forms heterodimers with TLR1 or TLR6	[11, 12, 13, 14, 15]
TLR3	Detects double‐stranded viral RNA	Dendritic cells, NK cells, various immune cells and fibroblasts	Forms homodimers	[16, 17, 18]
TLR4	Detects lipopolysaccharides from gram negative bacteria	Macrophages, dendritic cells, endothelial cells, adipocytes, hepatocytes, skeletal muscle, pancreatic β cells, and epithelial cells	Forms homodimers	[13, 19, 20, 21, 22, 23]
TLR5	Identifies flagellin found in bacterial flagella	Intestinal epithelial cells, monocytes, NK cells, T cells, and dendritic cells.	Forms homodimers	[24, 25]
TLR6	Identifies gram‐positive bacteria and fungi diacyl lipopeptides	Monocytes, macrophages, and B cells.	Forms heterodimers with TLR2	[26, 27]
TLR7	Detects single stranded viral RNA	Plasmacytoid dendritic cells, B cells, and to a lesser extent macrophages	Forms homodimers	[26, 28]
TLR8	Detects single‐stranded viral RNA	Monocytes, macrophages, and lower levels in NK cells.	Forms homodimers	[28]
TLR9	Recognizes unmethylated CpG DNA from viruses and bacteria	Plasmacytoid dendritic cells, B cells and at lower levels in monocytes, NK cells, and T cells.	Forms homodimers	[26, 29, 30]
TLR10	Complete function not yet fully elucidated, suggested to elicit an anti‐inflammatory response	Highly expressed in B cells, low or undetectable in monocytes, NK cells and T cells.	Forms either heterodimers with TLR2, TLR6, TLR1, or homodimers	[31, 32]

Without stimulation, TLRs are predominantly expressed on the cell membrane (TLR1, TLR2, TLR4–6 and TLR9‐TLR12) or in endosomes (TLR3, TLR7, TLR8, and TLR9) [8]. One of the best characterized and well‐studied TLRs is TLR4, also known as cluster of differentiation 284 (CD284). TLR4 is implicated in the defense response against both gram negative and gram‐positive bacteria, mycobacteria, some viruses, and yeast [33]. Additionally, TLR4 signaling has been linked to several different metabolic conditions, including obesity, diabetes, and cardiovascular diseases (CVDs) [34, 35]. This has been attributed to its role in the regulation of inflammation and metabolism, whereby chronic TLR4 activation leads to chronic low‐grade inflammation, a hallmark of these metabolic diseases [36]. This review will focus on the role of TLR4 in inflammation, glucolipid metabolism, and metabolic disorders.

## 2 Obesity and Its Development

Although multiple factors contribute to the development of obesity, the main takeaway is that obesity develops when sustained energy intake is greater than energy expenditure, resulting in the expansion of adipose tissue (AT) [37]. Historically viewed as a passive energy storage site, research has shown that AT acts as an active endocrine organ that secretes bioactive molecules, such as adipokines, which regulate systemic metabolism [38]. With the progression of obesity, adipocytes experience hypertrophy and hyperplasia, after storage capacity is exceeded, lipids begin to accumulate in visceral depots and in non‐AT, such as in the liver, skeletal muscle, and pancreas [39]. Hypertrophic adipocytes become metabolically stressed and hypoxic, which increases the rate of lipolysis, releasing free fatty acids (FFAs) and danger signals into the AT microenvironment and circulation [40].

Consequently, the release of FFAs and danger signals activates and recruits immune cells, particularly pro‐inflammatory macrophages, which create crown like structures around apoptotic adipocytes [41]. This shift to pro‐inflammatory adipokine profile and activated immune cells establishes a state of chronic low‐grade inflammation [42]. In this context, endogenous ligands, such as saturated fatty acids (SFAs) and circulating endotoxins, interact with pattern recognition receptors, including TLR4, activating NF‐κB and MyD88 pathways that sustain metabolic inflammation and instigate insulin resistance [43]. In parallel, TLR4 activation in adipocytes has been shown to enhance de novo lipogenesis by upregulating lipogenic transcription factors and enzymes, thereby promoting triglyceride accumulation in AT, further aggravating obesity related metabolic dysfunction [44, 45]. This framework provides a potential mechanistic link between obesity, inflammation, and TLR4 that is explored in the following sections.

## 3 Activation of TLR4 and Its Role in Inflammation

TLR4 is activated through the identification and detection of PAMPs, particularly lipopolysaccharides (LPS) [46]. However, in order for TLR4 to instigate a signaling cascade, it requires association with adapter proteins, such as CD14 or myeloid differentiation 2 (MD2), located on the cell surface [47]. Once TLR4 recognizes and binds to LPS, this induces its activation and the formation of TLR4‐MD2‐LPS complexes, which can homodimerize and induce the signaling of two distinct pathways [13, 48].

**FIGURE 1:**
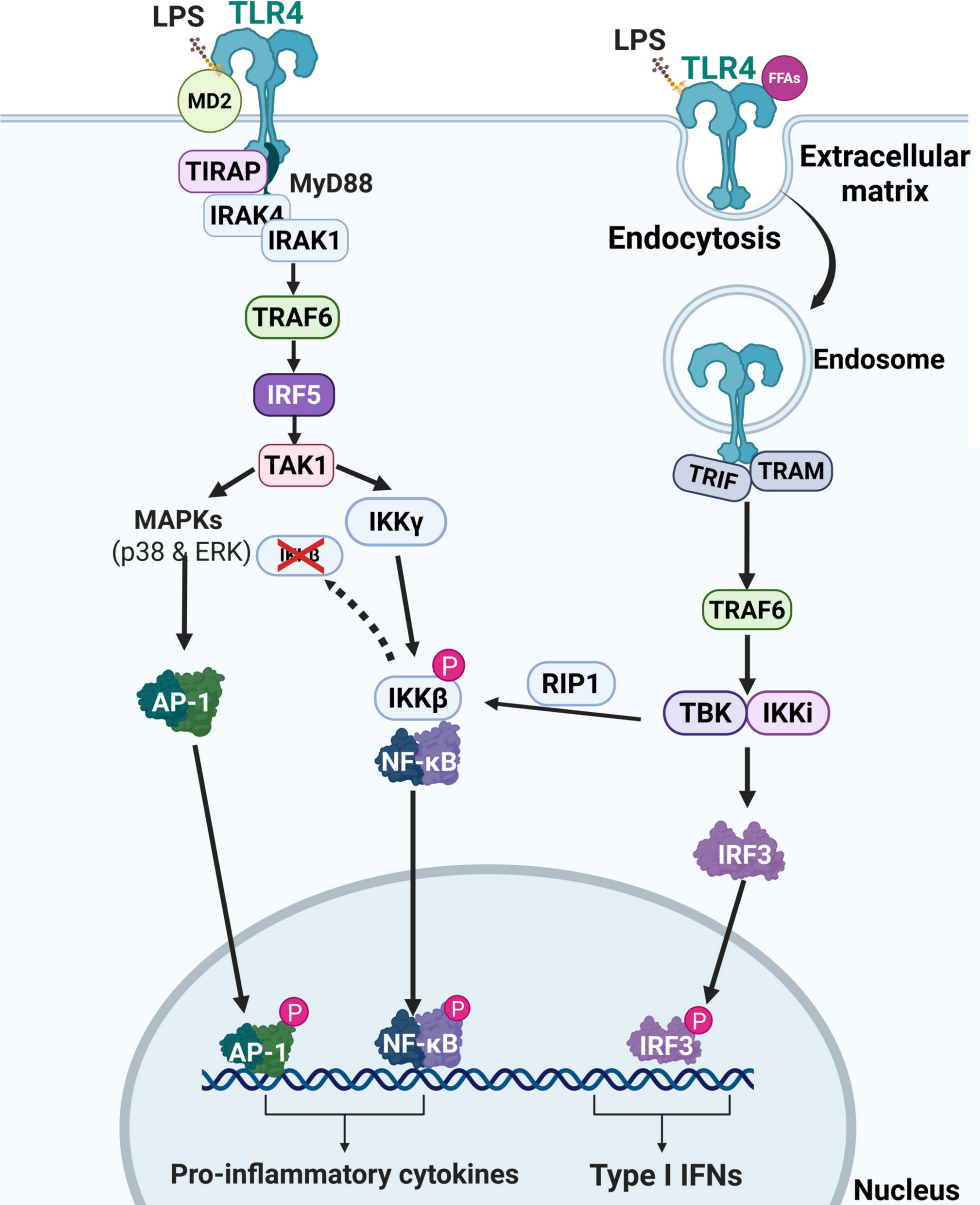
TLR4 signaling pathway. The figure demonstrates two distinct TLR4 signaling pathways initiated by TLR4 activation via lipopolysaccharides (LPS) or FFAs. The first pathway is the MyD88 (myeloid differentiation primary response 88) dependent pathway. Ligand binding to the TLR4/MD‐2 complex localized to the plasma membrane on the cellular surface instigates the recruitment of adaptor proteins such as MyD88 via TIRAP (toll/interleukin‐1 receptor domain–containing adaptor protein). This causes the activation of IRAK1/4 (IL‐1 receptor associated kinases) and, subsequently, activating TRAF6 (tumor necrosis factor receptor associated factor 6), prompting downstream signaling cascades. TRAF6 is able to activate MAPKs (such as ERK and p38), resulting in the transcription of AP‐1. Moreover, TRAF6 activates IKK complexes (IKKγ and IKKβ), which initiates the transcription of NK‐κB. The transcription of both AP‐1 and NF‐κB promote the transcription of proinflammatory cytokines. The second pathway is the MyD88 independent pathway, aka the TRIF (TIR‐domain–containing adapter inducing interferon β) dependent pathway. Following ligand binding, TLR4 undergoes endocytosis, where TLR4 interacts with TRIF and TRAM (TRIF‐related adaptor molecule). TRIF is able to recruit TRAF6, TBK (TANK‐binding kinase 1) and IKKi to activate IRF3 (interferon regulatory factor 3), promoting the transcription of type 1 interferons. Figure created with http://BioRender.com.

TLR4 distinguishes itself from other TLRs by its ability to activate two distinct signaling pathways. One pathway is the “traditional pathway” which is activated by TIRAP (toll/interleukin‐1‐receptor [TIR]‐domain–containing adaptor protein) and MyD88, which induce the synthesis and production of pro‐inflammatory cytokines. The second pathway is stimulated by the adaptors TRIF (TIR‐domain–containing adaptor protein inducing interferon‐β) and TRAM (TRIF‐related adaptor molecule), which activates the production of type I interferons [49]. Activation of either the MyD88‐dependent or MyD88‐independent pathways leads to the activation of lymphocytes, the expression and the upregulation of stimulatory signals, as well as the subsequent release of proinflammatory cytokines and chemokines [33]. The proinflammatory cytokines stimulated by TLR4 activation include IL‐6, IL‐1β, and TNF, which promote the recruitment of more immune cells to the site of injury or infection (Figure 1) [50].

Research has highlighted the role of TLR4 in various autoimmune and metabolic disorders [51, 52]. The dysregulation of TLR4, and its subsequent signaling pathways, induces chronic inflammation, which has been recognized as a hallmark of autoimmune conditions, such as systemic lupus erythematosus (SLE) [53] and rheumatoid arthritis [54]. Moreover, imbalance of TLR4 activity has also been implicated in cancer, as well as metabolic conditions, such as obesity and T2D.

## 4 Role of TLR4 in Obesity‐Associated Inflammation

Obesity is characterized by chronic low‐grade inflammation, driven in part by TLR4, contributing to obesity related insulin resistance [55]. Supporting this, several studies have shown that TLR4 is upregulated in the AT of individuals with obesity [56, 57], and that TLR4 knockout mice 4 are more likely to be protected from diet‐induced insulin resistance and inflammation [45]. The proposed belief is that TLR4 may be stimulated by endogenous ligands, particularly SFAs [58].

FFAs, specifically SFAs, were initially reported to induce the activation of TLR4, a concept partially based on the structural similarities between LPS and FFAs [59]. On this basis, diets rich in FFAs were suggested to increase the stimulation of TLR4, with SFAs thought to be recognized by CD14‐TLR4‐MD2 complexes, triggering an inflammatory response [60, 61]. This can lead to modifications to gut microbiota, causing an overproduction of LPS, especially after high‐fat intake, and further inducing TLR4 activation. This is suggested to increase LPS, and subsequent endotoxemia, leading to increased oxidative stress, producing oxidative modifications of low‐density lipoproteins (oxLDL), and generating further inflammation via CD36‐TLR4‐TLR6 complexes [60]. Upon activation of TLR4, downstream pro‐inflammatory signaling pathways are triggered, including the nuclear factor kappa‐light‐chain‐enhancer of activated B cells (NF‐κB) pathways [62]. The activation of the NF‐κB pathway eventually leads to the production of pro‐inflammatory cytokines, such as IL‐6 and tumor necrosis factor alpha (TNF) [63].

However, this model has been challenged. Lancaster et al. suggests that, although TLR4 is required for SFA‐induced inflammation, it is not a direct receptor for SFAs [64]. The authors proposed that the SFA palmitate is not a direct agonist of TLR4 but instead stimulates a noncanonical and slower inflammatory pathway compared to the classical pathways triggered by the known TLR4 agonists, such as LPS [64]. Moreover, the authors infer that TLR4 activation by SFA may stem from LPS contamination with bovine serum albumin (BSA) in experimental reagents that activate TLR4 [64]. Collectively, these findings support a more nuanced view in which SFAs may not be direct ligands of TLR4 but rather act as modulators and amplifiers of TLR4‐dependent inflammation.

Recently, many studies have shown evidence of a link between gut microbes and the dysregulation seen in obesity [65]. These results demonstrate that high‐fat diets (HFDs) can alter gut bacterial taxa by increasing gram‐negative bacteria, which release LPS [66]. Increased LPS can disrupt intestinal epithelial cells, leading to dysfunction in tight junction genes and increased gut permeability, allowing LPS to enter the circulation [66]. The circulating LPS activates the innate immune system via activation of TLR4, and subsequent downstream signaling of MyD88/NF‐κB pathways [67, 68]. This LPS‐TLR4 axis links diet‐induced changes in the gut microbiome to insulin resistance and obesity. Interestingly, studies are also reporting that changes in the gut microbiome may also influence the blood brain barrier, subsequently leading to neuroinflammation. Huwart et al. showed that TLR4 knockout mice fed an HFD were partially protected from HFD–induced neuroinflammation and in food reward dysregulation [69].

Of note, it has also been reported that changes to the gut microbiota and TLR4 signaling surpasses classical metabolic tissues [70]. In fact, there have been reports that linked gut dysbiosis induced by HFD in promoting testicular inflammation and dysfunction [70]. This implicates innate immunity, specifically pathways involving TLR4, in obesity‐related male reproductive organ impairment. Comparably, newly emerged data from HFD models show sexually dimorphic TLR4 responses in the brain and periphery, macrophage and microglia‐specific TLR4 deletion supports greater protection against diet‐induced neuroinflammation and metabolic disturbances in females than males, suggesting a sec‐specific TLR4 signaling in obesity driven neuroinflammation [71].

## 5 TLR4 and Obesity‐Associated Adipose Thermogenesis

**FIGURE 2:**
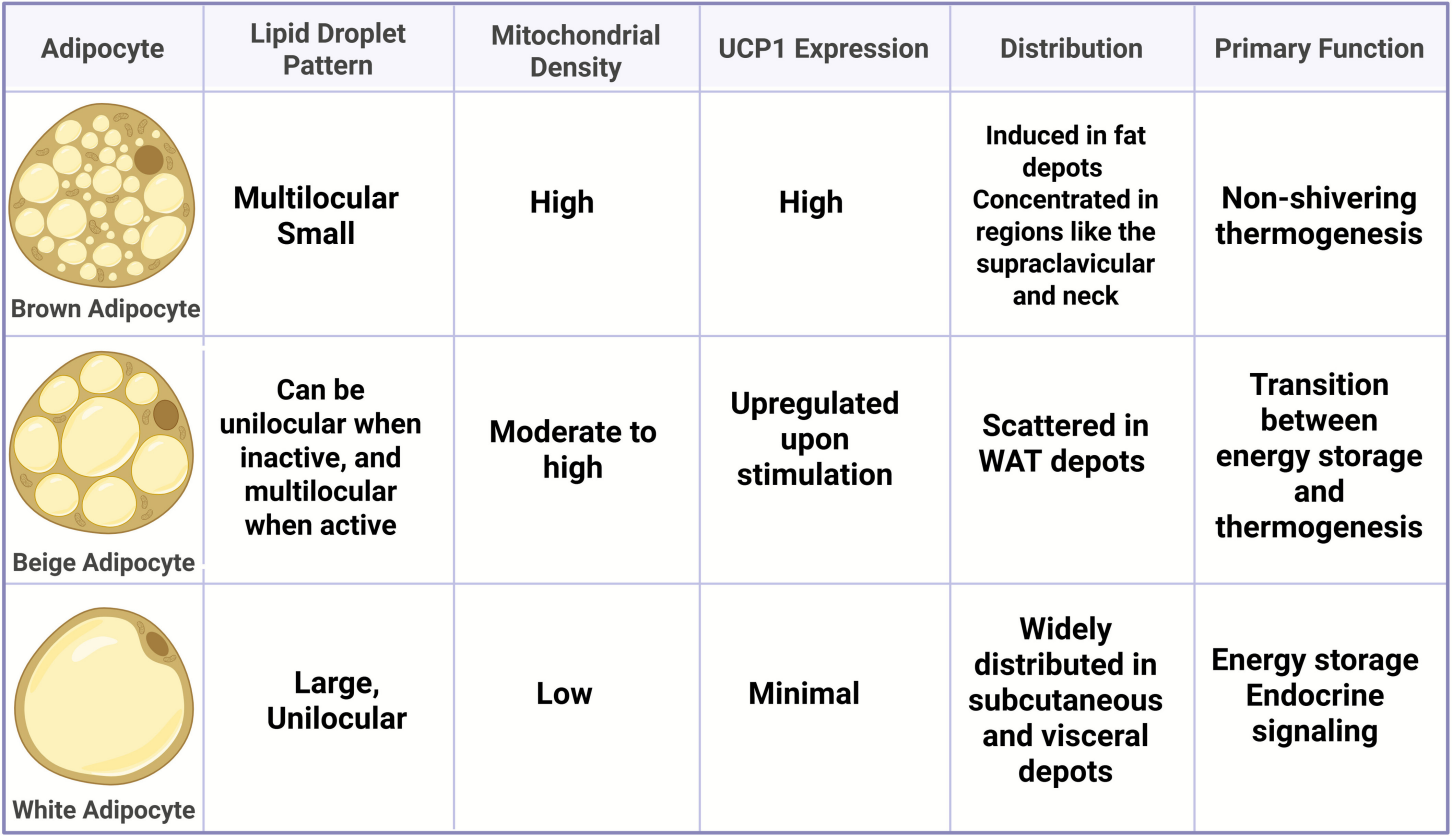
Side‐by‐side schematic comparison of white, beige, and brown adipocytes. This figure highlights key differences in lipid droplet pattern, mitochondrial density, UCP1 expression, tissue distribution, and primary function. Figure created with http://BioRender.com.

Thermogenesis is the process by which chemical energy is converted into heat as an adaptive response to cold exposure. There are two main kinds of AT directly involved in thermogenesis, these are brown (BAT) and beige AT. BAT is a specialized form of AT that is believed to be the main site of thermogenesis and energy expenditure facilitated by uncoupling protein 1 (UCP1) [72]. Beige AT are *brown‐like* adipocytes located within WAT deposits. Beige AT, besides their location, present with similar morphological and functional characteristics as BAT. The expression of thermogenic genes, namely, UCP1, is prevalent in beige tissue, with some reports suggesting that the levels may be able to reach that of BAT [73]. Figure 2 shows a schematic comparison of the different adipocytes.

**FIGURE 3:**
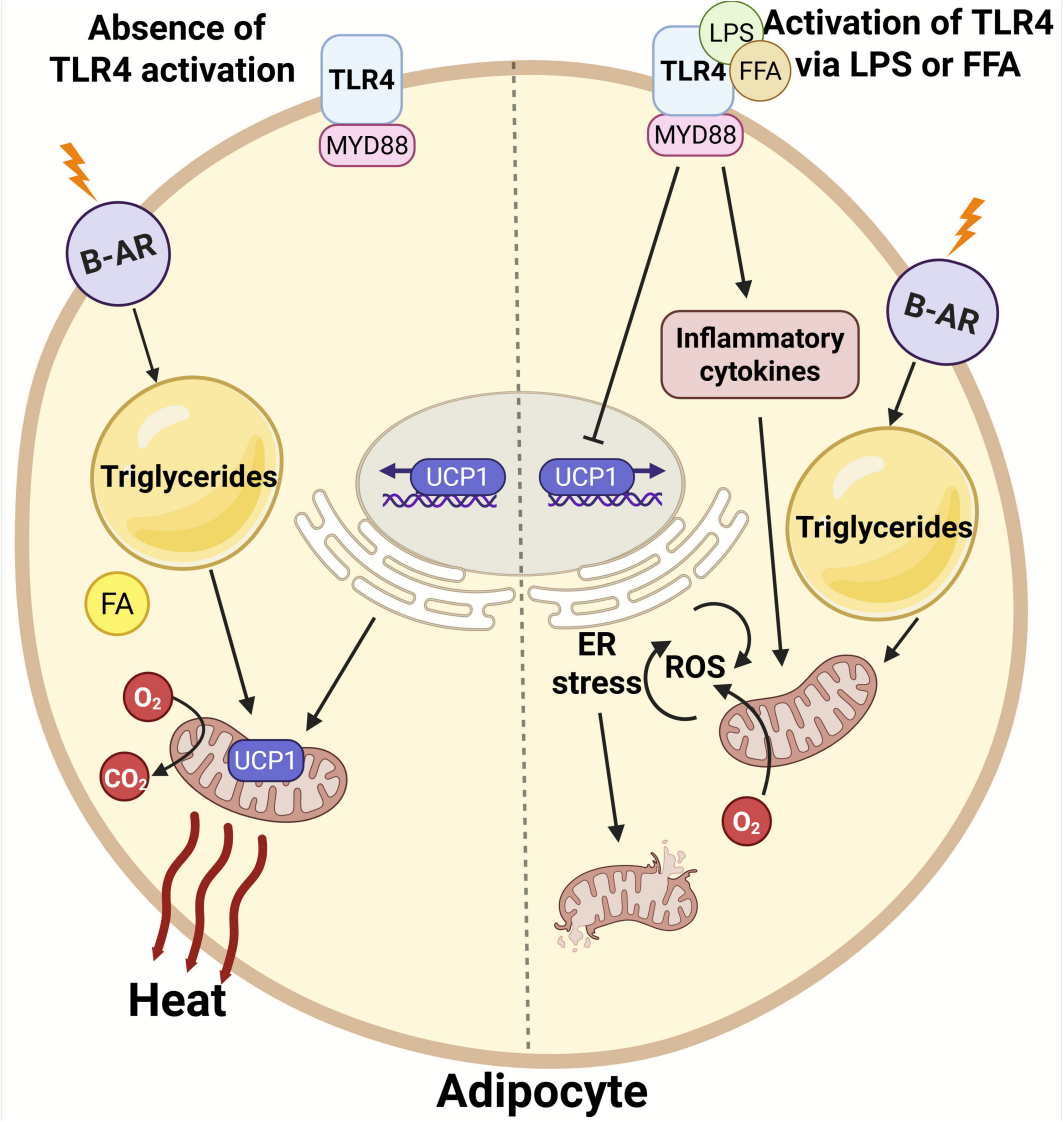
The proposed role of TLR4 in adaptive thermogenesis in adipocytes. This figure depicts the impact of adaptive thermogenesis in the presence or absence of TLR4 activation via lipopolysaccharides or FFAs. **Left panel:** In the absence of TLR4 activation, β‐adrenergic receptors (β‐AR) are stimulated in response to cold stimulation induces lipolysis, causing the breakdown of triglycerides into FFAs. These FFAs are then utilized by mitochondria for β‐oxidation and energy. Within brown adipocytes, mitochondria activate uncoupling protein 1 (UCP1), facilitating proton leakage across the mitochondrial membrane, which leads to the generation of heat, that is, thermogenesis. Normally, this process maintains metabolic homeostasis, with minimal inflammation and stress within the cell. **Right panel:** In the presence of TLR4 activation by LPS or FFA, an inflammatory milieu is initiated through a MyD88 dependent pathway. This TR4 activation disturbs normal cell processes and promotes the generation of reactive oxygen species (ROS) and endoplasmic reticulum (ER) stress. This inflammatory milieu downregulates the expression of UCP1, impairing mitochondrial function and thermogenesis. As a consequence, energy dissipation as heat is reduced, further contributing to metabolic dysfunction and the potential development of obesity or obesity related disorders. This figure was adapted from Okla et al. (2015) [76]. Figure created with http://BioRender.com.

In obesity, the mechanism by which chemical energy is converted into heat seems to be compromised. Although the precise molecular processes impacted have not been fully elucidated, studies suggest that TLR4, and its role in inflammation, may play a part in the loss of thermogenic function in individuals with obesity [74]. Mitochondria play a critical role in producing cellular energy and reactive oxygen species (ROS), which are necessary for oxidative stress and TLR4‐mediated immune activation [75]. Chronic TLR4 stimulation, via LPS or FFAs, has been shown to significantly impair mitochondrial respiration (Figure 3). This was also observed in mouse skeletal muscle, where TLR4 deficiency impaired mitochondrial function [77]. A study by Okla et al. demonstrated that mice fed an HFD expressed higher levels of TLR4 compared with mice fed a low‐fat diet, this TLR4 activation was associated with increased levels of ROS that instigated endoplasmic reticulum (ER) stress and subsequent mitochondrial dysfunction [76]. In fact, researchers have found that the deletion of TLR4 within hematopoietic cells was able to improve the homeostasis in perivascular AT, subsequently decreasing TNFα release by macrophages and increasing mitochondrial function in BAT [78]. Together, these findings support a peripheral role for TLR4 in shaping AT inflammation, mitochondrial health and thermogenic capacity.

In addition to these peripheral mechanisms, central TLR4 signaling also influences BAT activity. Because of wide tissue distribution of TLR4, studies have found that TLR4 deficient pro‐opiomelanocortin neurons (POMC) were able to modulate AT browning, and subsequent thermogenesis in a sex dependent manner [79]. Li et al. reported that a specific deletion of TLR4 in POMC neurons was sufficient to increase thermogenesis and energy expenditure, leading to reduction of body weight in male mice [79]. These data indicate that central neuronal TLR4 and peripheral TLR4, localized to AT and immune cells, act in a complementary manner. Meanwhile, neuronal TLR4 controls sympathetic input to BAT and adipose or immune cell TLR4 regulated local inflammatory and mitochondrial responses within adipose depots. This further highlights the importance of TLR4 signaling in attenuating adaptive thermogenesis.

## 6 TLR4 in Insulin Resistance and T2D

A sustained pro‐inflammatory state has been implicated in the development of insulin resistance in numerous studies [80]. Inflammation has been associated with reducing insulin sensitivity, and insulin secretion, via the continued activation of signaling pathways that directly interfere with the insulin signaling pathway. It is important to note that TLR4 is expressed not only in classical innate immune cells, but also in metabolically relevant tissues, including adipocytes, hepatocytes, skeletal muscle and pancreatic β‐cells, as well as in brain‐resident cells such as microglia [81, 82]. Moreover, because of the suggested role of TLR4 as a molecular link that connects nutrients, such as lipids, and inflammation to the innate immune system, it may participate in the regulation of energy and insulin resistance as a response to changes in the nutritional status. Studies, such as Shi et al. have demonstrated that FFAs, which result from a HFD, can activate TLR4 in both adipocytes and macrophages, inducing an inflammatory response [83].

**FIGURE 4:**
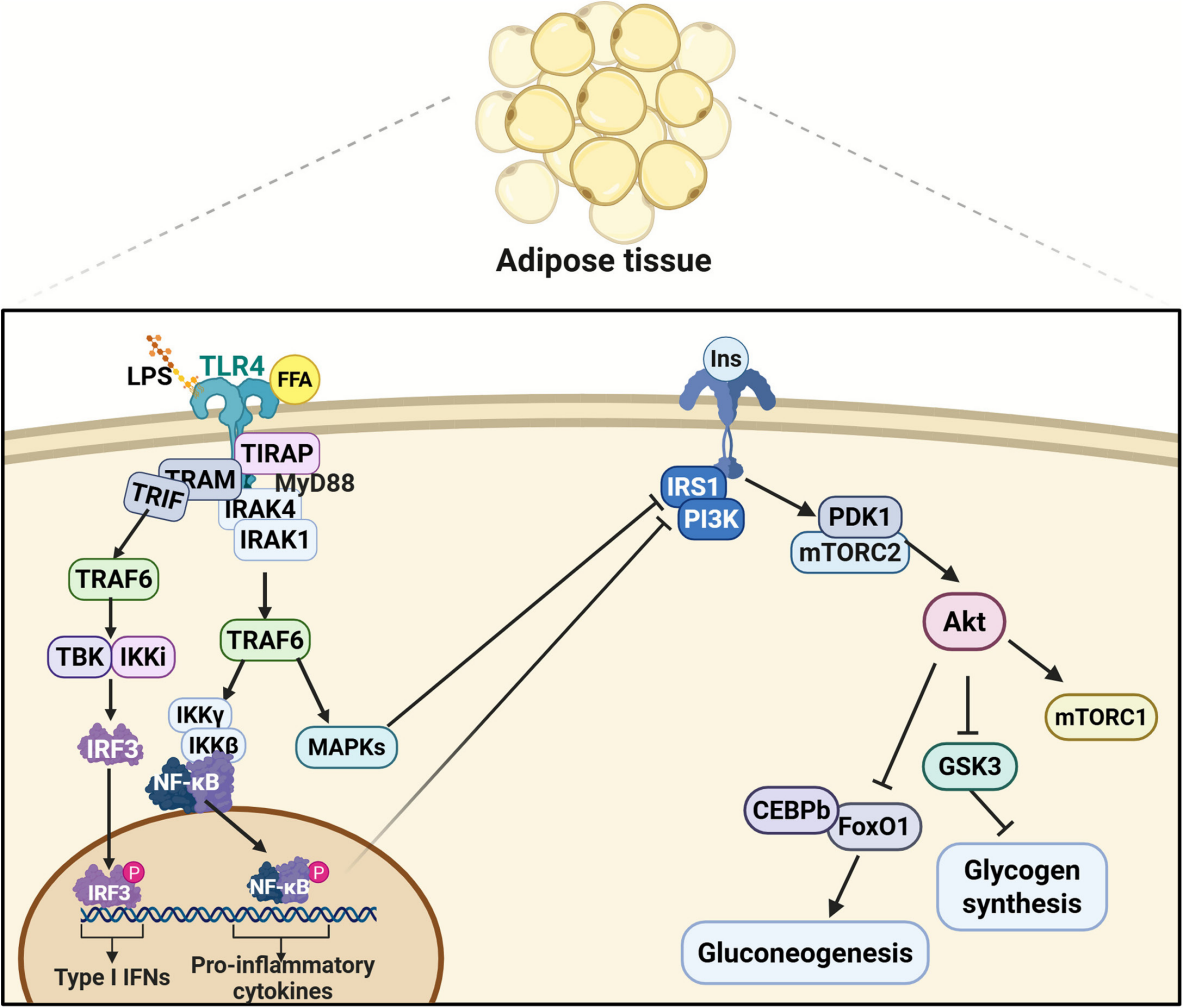
Interaction between the TLR4‐mediated inflammatory pathway and the insulin signaling pathway within adipose tissue. TLR4 signaling pathway (left): activation of TLR4 by lipopolysaccharides (LPS) or FFAs triggers two distinct pathways, TRIF‐dependent pathway or the MyD88‐dependent pathway: involves MyD88 and TRAF6, resulting in the activation of NF‐κB via IKKβ and the MAPK pathway, both of which promote the transcription of pro‐inflammatory cytokines. These inflammatory mediators contribute to local and systemic inflammation, disrupting metabolic homeostasis in adipose tissue. Insulin signaling pathway (right): insulin binds to its receptor, activating IRS1 and PI3K, which phosphorylate and activate downstream effectors, including Akt: regulates glucose homeostasis by promoting glycogen synthesis (via inhibition of GSK3) and suppressing gluconeogenesis (via inhibition of FoxO1 and CEBPβ) and mTORC1/mTORC2: play roles in lipid metabolism, cell growth, and nutrient sensing. Chronic activation of TLR4 signaling induces inflammatory cytokines and oxidative stress, which impair IRS1 phosphorylation and Akt activation. This results in reduced insulin signaling efficacy, contributing to insulin resistance and metabolic dysfunction in adipocytes. This emphasizes how inflammation driven by TLR4 activation disrupts insulin signaling, a key mechanism underlying obesity‐related metabolic diseases such as T2D. Figure created with http://BioRender.com.

Conversely, Tao et al. suggested a dichotomous role for TLR4 signaling in obesity and insulin sensitivity. They proposed that TLR4 has a dual role in normal adipocyte function and adipocyte response to HFD. Chronic HFD exposure in adipocyte‐specific TLR4 knockout mice (Tadipo mice) supported healthy adipose development and promoted insulin sensitivity, whereas acute exposure with SFAs promoted insulin resistance in these mice [84]. Hence, it can be extrapolated that TLR4 may not directly influence insulin resistance in obese animals, or in response to a HFD. Research has suggested that retinol binding protein‐4 (RBP4) can activate TLR4 and induce proinflammatory pathways that contribute to insulin resistance. Moraes‐Vieira et al. demonstrated, through the use of RBP4−/− knockdown mice, which RBP4 is able to mitigate macrophage inflammation via MyD88 and mitogen‐activated protein kinase (MAPK) pathways, which subsequently induce NF‐κB. This leads to the phosphorylation of key signaling proteins, such as c‐Jun N‐terminal kinase (JNK), p38 in the MAPK pathway, and ERK [85]. Similarly, TLR4 activation can induce the JNK pathway and p38, both pathways can cause the phosphorylation of serine residues localized on insulin receptor substrate‐1 (IRS‐1) protein. This, in turn, inhibits the role of IRS‐1 and diminishes the downstream pathways involved in insulin signaling, causing reduced glucose uptake and insulin resistance (Figure 4) [81, 86].

Beyond AT, skeletal muscle is a major site of insulin‐mediated glucose uptake; therefore, impaired skeletal muscle metabolism significantly affects whole body glucose regulation and insulin sensitivity [87]. Studies have shown that human myotubes express TLRs 1–6 and functionally respond upon activation of these receptors. Activation of TLR3–6, including TLR4, in myotubes alters glucose and fatty acid metabolism and myokine secretion, such as IL‐6, IL‐8, and granulocyte macrophage colony‐stimulating factor (GM‐CSF) [23]. Further studies have observed that increased levels of ceramides, which are involved in FA oxidation regulation and are able to impact insulin signaling through the protein kinase B/AKT signaling pathway, diminishes muscle glucose usage [88]. The binding of FA to TLR4 is suggested to trigger the synthesis and collection of ceramides via the instigation of inflammatory pathways [88]. These findings support a critical role of muscle TLR4 signaling in mediating FFA‐induced defects in insulin action.

The liver is equally critical for systemic insulin sensitivity, due to its role in glucose production and lipid metabolism [89]. Both Kupffer cells (KCs) and hepatocytes express TLR4, with TLR4 deletion in mice showing improved glucose tolerance and insulin sensitivity while reducing hepatic steatosis and inflammatory gene expression under HFD feeding [45]. Global or hepatocyte‐specific TLR4 knockout attenuates diet‐induced hepatic steatosis, ER stress, and inflammatory signaling, and these findings indicate that hepatic TLR4 is a key driver of liver insulin resistance and metabolic dysfunction [45, 90, 91].

## 7 TLR4 in the Liver and in Metabolic Dysfunction Associated Steatotic Liver Disease

The liver is a central organ for both metabolism and immune surveillance, constantly exposed to gut‐derived antigens and microbial products through the portal vein. This unique functional position makes it highly relevant for innate immune receptors like TLR4, which recognizes LPS from gram‐negative bacteria. Dysregulation of TLR4 signaling has been implicated in liver diseases, particularly in the progression of healthy liver through the stages of the metabolic dysfunction associated steatotic liver disease (MASLD). MASLD represents a spectrum of conditions affecting the liver, ranging from relatively benign steatosis to fibrosis and inflammation in the progressive form of steatohepatitis (MASH). Given the liver's constant interaction with microbial products, it is important to consider how TLR4 expression varies among its different cell populations and how it contributes to disease mechanisms.

Considering the whole tissue, TLR4 mRNA expression is relatively low in the healthy liver when compared to other organs, suggesting a higher tolerance of the liver to TLR activation from endogenous ligands [92]. However, activation of TLR4 in the liver in response to endogenous or exogenous ligands associated with injury, such as viral hepatitis, alcoholic or nonalcoholic liver disease, and liver autoimmune disease, can induce chronic inflammatory processes and alter lipid metabolism, impacting overall tissue function [93]. Persistent activation of TLR4, and the subsequent chronic inflammation, can lead to liver fibrosis and steatosis [92].

Studies found that silencing TLR4, via lentiviral‐packed siRNA transfection, alleviates systemic and hepatic inflammation in mice, while additionally ameliorating high‐fat‐diet–induced obesity and insulin resistance [94]. Furthermore, a study by Fu et al. demonstrated that treatment with a selective inhibitor of histone deacetylation 6, ACY‐1215, had a protective impact on a cellular model of nonalcoholic fatty liver disease (NAFLD) (now redefined as MASLD) by inhibiting the TLR4/MyD88/MAPK/CD14/NF‐κB signaling pathway [95].

When considering component cells of the liver, TLR4 is expressed at varying levels between hepatocytes and liver nonparenchymal cells (NPCs), such as KCs, liver sinusoidal endothelial cells (LSECs), hepatic stellate cells (HSCs), and monocyte‐derived macrophages (MoMFs) (Table 2).

**TABLE 2:** Expression of TLR4 in liver component cells and regulation in MASLD.

Cell type	Relative TLR4 expression	Known function and role of TLR4	Adaptation in MASLD
Hepatocytes	Low (upregulated in disease)	Induces oxidative stress and lipogenesis upon TLR4 activation	Promotes lipid accumulation and liver injury (de novo lipogenesis)
Kupffer cells (KCs)	High [96]	Major immune sentinels, produce pro‐inflammatory cytokines upon LPS activation	Contributes initially to chronic inflammation by releasing cytokines and chemokines, can interact with HSC and contribute to fibrosis
Monocyte‐derived macrophages (MoMFs)	High	Recruited in response to liver injury, amplify inflammation	Contributes after recruitment to chronic inflammation (by releasing cytokines and chemokines), interact with HSC and contribute fibrosis
Liver sinusoidal endothelial cells (LSECs)	Moderate to high [96]	Detect LPS, regulate immune response	Dysfunction contributes to inflammation and fibrosis
Hepatic stellate cells (HSCs)	Moderate	Activated by TLR4 signaling to produce extracellular matrix	Leads to liver fibrosis and progression to cirrhosis

Although hepatocytes exhibit low basal TLR4 expression compared to immune cells [97], it is increased in pathological conditions including MASLD and MASH. TLR4 activation in hepatocytes promotes oxidative stress, lipid accumulation, and lipogenesis. A recent study showed that the LPS/TLR4 axis regulates hepatic glutaminase (GLS1) expression, contributing to hepatic ammonia accumulation during the progression from steatosis to MASH [98]. In a study of mice with hepatocyte‐specific deficiency of TLR4 (TLR4 KO), loss of TLR4 leads to improved glucose tolerance, enhanced insulin sensitivity, and ameliorated hepatic steatosis despite weight gain on an HFD [45].

KCs, resident liver macrophages, are the primary immune sentinels in the liver and express high levels of TLR4. They respond to LPS by producing pro‐inflammatory cytokines (TNF, IL‐6, IL‐1β), which can exacerbate liver damage and insulin resistance. In disease conditions, infiltrating MoMFs also upregulate TLR4 and contribute to inflammation and fibrosis. The interplay between KCs and MoMFs is crucial in the pathogenesis of MASH, with TLR4 signaling playing a central role [99].

Macrophages require TLR4 to reveal steatosis, inflammation, and fibrosis to different degrees in murine models of MASH (in this study using the choline‐deficient amino acid defined diet; CDAA) [100]. This was demonstrated by grafting TLR4−/− or wild‐type bone marrow into mice that were already either competent for or that lacked TLR4 in the whole body, effectively reconstituting TLR4 in the myeloid compartment of TLR4 KO mice or depleting it in wild‐type mice. Grafting TLR4‐deficient bone marrow resulted in lower plasma transaminases following CDAA diet on both wild‐type and TLR4 KO backgrounds. Steatosis, inflammation, and fibrosis were altered to different degrees in different chimeric combinations, with TLR4‐deficient bone marrow transplantation into TLR4 KO mice being the most protected. This indicates that regulation of each process is intricate and relies on effective communication between cells, downstream of their own expression of TLR4 [100].

LSECs are highly specialized endothelial at the interface between the blood and hepatic parenchyma. They express TLR4 and contribute to immune surveillance by detecting bacterial products and modulating inflammatory responses. In a study, they show that stimulation of TLR4 in LSECs leads to the production of IL‐6 [96]. LSEC dysfunction has been linked to steatosis progression and increased fibrosis.

HSCs, which physiologically store vitamin A and regulate extracellular matrix production, express TLR4 and are activated by LPS. This activation promotes fibrogenesis, establishing a direct link between innate immunity and liver fibrosis. TLR4 activation in HSCs potentiates TGF‐β signaling, thereby amplifying liver fibrosis [101].

## 8 TLR4 and CVDs

Latest research in the implications of TLR4 in metabolic diseases also highlight the role of TLR4 in CVDs [102, 103]. TLR4 signaling has been implicated in the progression of atherosclerosis, thrombosis, myocardial infarction, and heart failure [102, 104, 105]. As previously stated, TLR4 is widely expressed. This wide range allows for the receptor to conduct its function in a plethora of tissues, including those of the myocardium.

Endothelial cells and atherosclerotic plaques have high expression levels of TLR4 [102]. Through its role in inflammation, TLR4 activation can lead to the expression of vascular cell adhesion molecules (VCAM‐1) and intercellular adhesion molecule 1 (ICAM‐1), facilitating the recruitment of leukocytes to the atrial wall [106]. This recruitment encompasses a vital early stage in the development of atherosclerosis. Moreover, TLR4 is a target protein of neutrophil elastase in vascular smooth muscle cells (VSMCs), where this receptor shows control over regulatory processes involved in VSMC proliferation via the TLR4/TRAF6/MyD88/IRAK1/NF‐κB pathway [107]. The vehement proliferation of VSMC encourages the formation of plaques [108]. Yin et al. demonstrated that ApoE−/− mice fed a HFD developed atherosclerotic plaques, however ApoE/TLR4−/− did not develop plaques. This study demonstrated that atherosclerotic plaques and VSMC foam cell formation occur in a TLR4 dependent manner [109].

Inflammation, concurrently, plays a vital role in the development of thrombosis [110]. Inflammation can activate coagulation, promoting thrombosis, and in turn thrombosis can further aggravate inflammation. Macrophages expressing TLR4 link inflammation and thrombosis via the activation of plasminogen activator inhibitor‐1 (PAI‐1) [48, 111, 112]. In vitro, PAI‐1 stimulates inflammation via the TLR4/NFκB pathway, in fact deletion of PAI‐1 resulted in a decreased inflammatory response and lower TLR4/NFκB pathway activity [113]. Moreover, inflammation can drive up the expression of PAI‐1, creating as cycle that links TLR4‐mediated inflammation and thrombosis development [48].

Studies have also shown that the inflammatory cascade seen in myocardial infarctions and coronary microembolism (CME) is partially driven by TLR4‐mediated activation of the MyD88/NF‐κB pathway [114, 115]. When CME occurs, often as a complication of percutaneous coronary intervention or acute coronary syndrome, it causes cellular damage and potential myocardial infarctions, which results in the release of DAMPS. These DAMPS then bind to and trigger TLR4 receptors localized to the surface of cardiac cells and infiltrating immune cells [115]. Subsequently, this leads to the downstream activation of the TLR4/MyD88/NF‐κB pathway, releasing NF‐κB, TNF‐α, IL‐1β, IL‐18, and NLRP3 inflammasome [116]. The induced inflammatory cascade promotes cardiomyocyte apoptosis, progressive cardiac dysfunction, and increased tissue damage in the affected area [116].

## 9 Human Evidence for TLR4 in Obesity and Metabolic Disease

Several studies have linked TLR4‐induced inflammation and obesity and obesity‐related complications. One study in individuals with obesity demonstrated a strong significant association of TLR2/TLR4 expression with TNF‐α and IL‐6 in peripheral blood mononuclear cells (PBMCs), suggesting a possible pathophysiological link between obesity and inflammation [56]. Furthermore, studies assessing the role of TLR4 in human colon carcinoma cells (SW480) deduced that TLR4 is, not only, expressed in SW480 cells, but may also promote tumor immune escape of human carcinoma cells by resisting apoptosis and enhancing immune‐suppressive factors [117], underscoring a broader relevance of TLR4‐mediated inflammation and human disease.

Additionally, it has been well documented that B cells are integral in initiating an immune response [118]. In terms of obesity, TLRs, particularly TLR2, TLR4, and TLR9 on the surface of B cells, are vital in adjoining innate and adaptive immune responses [118, 119]. Dysregulated TLR signaling on B cells is pivotal in disrupting immune homeostasis and has been associated with inflammation and AT dysfunction in individuals with obesity [119].

More recently, studies have attempted to divulge the role of polymorphisms in the TLR4 gene on responsiveness to LPS and susceptibility to inflammation. One such study by Rodríguez‐García et al. demonstrated that heterozygous single nucleotide polymorphisms (SNPs) in the TLR4 gene (−2081 G>A, 1196 C>T, and 896 A>G) conferred a higher risk of young adults developing obesity and altered lipid profiles [120], supporting a contribution of TLR4 genetic variation to human cardiometabolic risk.

## 10 Concluding Remarks and Future Perspectives

TLR4 has emerged as a key contributor to the pathogenesis of various metabolic syndromes, particularly those characterized by chronic, low‐grade inflammation such as obesity, insulin resistance, and T2D. Its heightened expression in metabolic tissues underscores its pivotal role in linking immune responses with metabolic dysfunction. Targeting TLR4 directly or modulating its downstream signaling pathways presents a promising therapeutic approach to reduce inflammation and improve metabolic outcomes. The involvement of specialized immune‐regulatory cells, such as LSECs, in expressing TLR4 further illustrates the complex interplay between immunity and metabolism and opens new avenues for targeted therapies. In obese AT, TLR4 regulates the secretion of adipokines and other inflammatory mediators, driving chronic inflammation and metabolic dysregulation. These insights support the potential development of TLR4‐specific inhibitors or biologics as novel treatment options. Clinically, such interventions could be used to complement existing therapies for metabolic disorders, particularly in patients with obesity‐driven inflammation who do not respond adequately to lifestyle or pharmacological interventions alone. Additionally, biomarkers associated with TLR4 activation may serve as diagnostic tools to identify individuals at risk for metabolic syndrome or monitor therapeutic responses. Continued translational research is essential to move TLR4‐targeted strategies from bench to bedside, potentially leading to more personalized and effective treatments for metabolic diseases.

## Conflicts of Interest

The authors declare no conflicts of interest.

## Data Availability

Data sharing not applicable to this article as no datasets were generated or analysed during the current study.
